# Central Nervous System Tuberculosis: An Imaging-Focused Review of a Reemerging Disease

**DOI:** 10.1155/2015/202806

**Published:** 2015-01-12

**Authors:** Morteza Sanei Taheri, Mohammad Ali Karimi, Hamidreza Haghighatkhah, Ramin Pourghorban, Mohammad Samadian, Hosein Delavar Kasmaei

**Affiliations:** ^1^Department of Radiology, Shohada-e-Tajrish Hospital, Shahid Beheshti University of Medical Sciences, Tehran, Iran; ^2^Department of Neurosurgery, Loghman Hakim Hospital, Shahid Beheshti University of Medical Sciences, Tehran, Iran; ^3^Department of Neurology, Shohada-e-Tajrish Hospital, Shahid Beheshti University of Medical Sciences, Tehran, Iran

## Abstract

Central nervous system (CNS) tuberculosis is a potentially life threatening condition which is curable if the correct diagnosis is made in the early stages. Its clinical and radiologic manifestations may mimic other infectious and noninfectious neurological conditions. Hence, familiarity with the imaging presentations of various forms of CNS tuberculosis is essential in timely diagnosis, and thereby reducing the morbidity and mortality of this disease. In this review, we describe the imaging characteristics of the different forms of CNS tuberculosis, including meningitis, tuberculoma, miliary tuberculosis, abscess, cerebritis, and encephalopathy.

## 1. Introduction

With the outbreak of acquired immunodeficiency syndrome (AIDS) and increasing frequency of other immunocompromising conditions in recent decades, tuberculosis has resurged and remained a major worldwide health problem. Although* Mycobacterium tuberculosis* can involve any organ, most commonly the lung, central nervous system (CNS) tuberculosis is the most devastating form of the disease. Approximately 5–10% of all patients with tuberculosis and up to 20% of patients with AIDS-related tuberculosis have CNS involvement [1–3].

CNS tuberculosis usually results from hematogenous spread, while direct spread from intra- or extracranial focus is rare [4]. The clinical and radiologic manifestations of CNS tuberculosis may mimic other infectious and noninfectious neurological conditions, such as brain tumors. Therefore, familiarity of infectious diseases specialists with the imaging presentations of CNS tuberculosis is essential for prompt and accurate diagnosis of this entity. Herein, we describe the different forms of CNS tuberculosis including meningitis, cerebritis, cerebral abscesses, tuberculomas, miliary tuberculosis, and spinal or calvarial involvement [5–7].

## 2. Tuberculous Meningitis

Meningitis is the most common manifestation of CNS tuberculosis which is most frequently seen in the children and adolescents [8, 9]. Tuberculous meningitis is mostly due to the hematogenous spread of* Mycobacterium tuberculosis*; however, it can also occur secondary to extension and/or rupture of a subpial or subependymal focus (i.e., Rich focus) to the subarachnoid spaces or into the ventricular system [10]. Tuberculous meningitis often has an insidious course with a nonspecific clinical presentation in early stages, especially in children. Therefore, the imaging plays a key role in the timely diagnosis and decreasing the morbidity and mortality.

Enhancing exudate in the basal cisterns is the most common and also a relatively specific manifestation of leptomeningeal tuberculosis on computed tomography (CT) and magnetic resonance (MR) images [11]. The exudate is composed of neutrophils, mononuclear cells, erythrocytes, and bacilli in the basal portions of the brain. Meningeal enhancement has been found in up to 90% of cases and is considered to be the most sensitive feature of tubercular meningitis [6, 11, 12]. The subpial exudate is primarily located in the inferomedial surface of the frontal lobes, the anteromedial surface of the temporal lobes, the superior aspect of the cerebellum, and the floor of the third ventricle [13]. Extension to suprasellar, interpeduncular, and pontomesencephalic cisterns may also occur from these primary sites [10]. In most cases, some degree of meningeal involvement is seen within the sulci over the cerebral convexities, the sylvian fissures, and also the ependymal surfaces of the ventricles; the latter usually occurs in the later stages of the disease [1, 14, 15].

**Figure 1:**
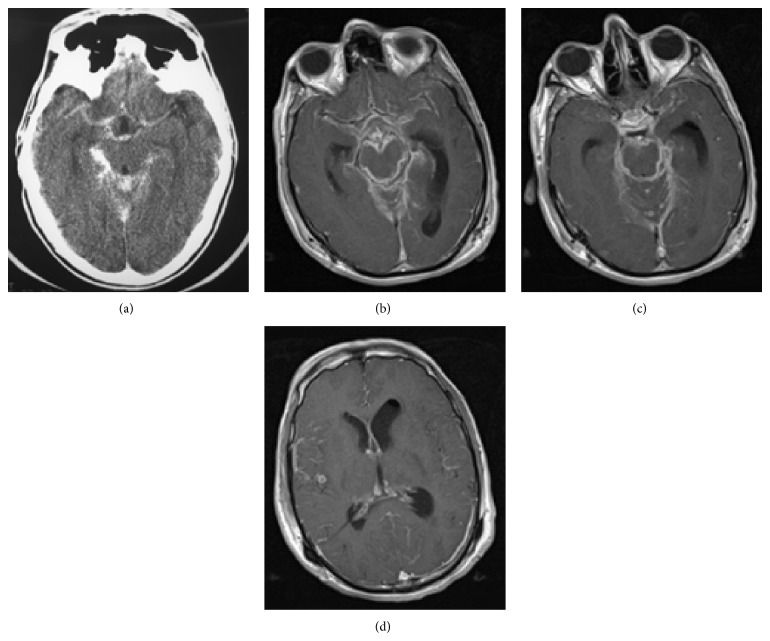
Meningeal tuberculosis. (a) Axial postcontrast brain CT shows typical thick enhancement of basilar cisterns. (b) Axial postcontrast T1-weighted MR images in a different patient ((b), (c), and (d)) demonstrate enhancing basilar exudates and leptomeningeal enhancement. A small tuberculoma in right temporal region (d) and hydrocephaly (more severe in left ventricle) and the evidence of prior craniotomy (Burr hole, left pneumoventricle) are also evident.

On CT images, the obliteration of the basal cisterns by isodense or mildly hyperdense exudates is the most common finding in tuberculous meningitis [1, 6, 13, 16]. The findings are better appreciated on MR imaging than on CT, especially on postcontrast MR images which show the enhancing cisternal exudates and leptomeningeal enhancement (Figure 1) [5]. Parmar et al. demonstrated that postcontrast fluid attenuation inversion recovery (FLAIR) images may have a higher specificity compared to contrast-enhanced T1-weighted images in detection of leptomeningeal enhancement [17]. Magnetization transfer spin echo imaging following contrast injection is superior to the conventional postcontrast imaging in demonstrating meningeal inflammation [7]. In later stages, there may be widening of subarachnoid spaces.

A similar pattern of meningeal enhancement may be seen in other infective meningitis, inflammatory diseases such as rheumatoid arthritis, sarcoidosis, or carcinomatous meningitis [7].

Other radiologic manifestations of tuberculous meningitis may be related to its possible complications, including progressive hydrocephalus, vasculitis, infarction, and cranial neuropathies [6, 7].

Communicating hydrocephalus, which is considered the most common complication of tuberculous meningitis, is usually secondary to the obstruction of cerebrospinal fluid (CSF) flow in the basal cisterns [1, 4, 6, 7]. In some cases, the hydrocephalus may be noncommunicating, resulting from obstruction due to tuberculoma or rarely tuberculous abscess.

**Figure 2:**
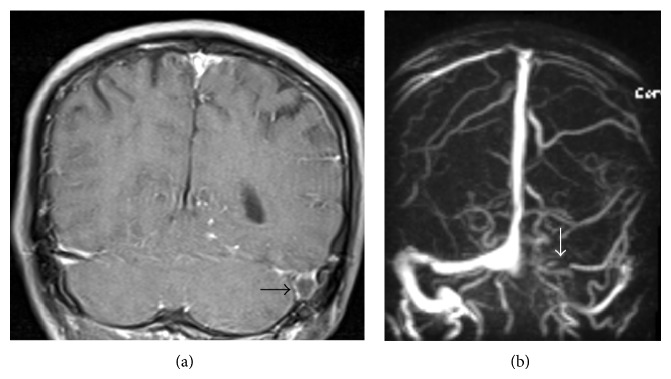
Dural venous sinus thrombosis as an only imaging evidence of tuberculous meningitis in a 45-year-old male who presented with headache and cerebrospinal fluid PCR positive for* Mycobacterium tuberculosis*. (a) Coronal postcontrast T1-weighted MR image demonstrates a filing defect within dilated left sigmoid sinus (*black arrow*). (b) MR angiogram reveals nonvisualization of transverse and sigmoid sinuses in left side (*white arrow*).

Ischemic infarct is also a common complication, being detected in 20–41% of patients on CT, mostly within the basal ganglia or internal capsule regions and resulting from vascular compression and occlusion of small perforating vessels (necrotizing arteritis) [14, 18, 19], particularly the lenticulostriate and thalamoperforating arteries, vessels which perfuse the so-called medial tuberculosis zone [16]. Tuberculous meningitis may also cause dural venous sinus thrombosis with resultant hemorrhagic infarct. Rarely, tuberculosis may present as isolated dural venous sinus thrombosis without any evidence of meningitis or its complications (Figure 2).

Cranial nerve involvement occurs due to vascular compromise, ischemia, or nerve entrapment in the basal exudates in 17–40% of cases, most commonly affecting the second, third, fourth, and seventh cranial nerves [13, 15]. The affected cranial nerves are best evaluated by MRI, where they may appear thickened, especially in their proximal segments, with high signal intensity on T2-weighted images and marked enhancement on postcontrast images.

## 3. Parenchymal Tuberculosis

Parenchymal disease may be isolated or associated with tuberculous meningitis. Parenchymal involvement usually presents as tuberculoma. It can also manifest as cerebritis, cerebral abscess, miliary tuberculosis, or tuberculosis encephalopathy.

### 3.1. Cerebritis and Cerebral Abscess

Parenchymal tuberculosis may occur with or without accompanying meningitis. Tuberculosis cerebritis or abscess may have an appearance similar to that of pyogenic bacterial infection on neuroimaging studies.

Focal tuberculous cerebritis is very rare [5], with hypo- and hypersignal intensities on T1- and T2-weighted images, respectively, and causes small areas of patchy enhancement on postcontrast images.

**Figure 3:**
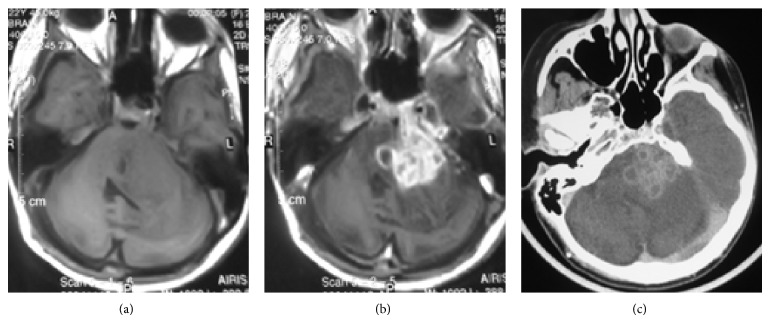
Tuberculous abscess mimicking a cerebellopontine angle tumor in a 22-year-old female with miliary pulmonary tuberculosis, right hemiparesis, left facial paresis, and sixth and seventh cranial nerves involvement. Axial T1-weighted image (a) shows a predominantly isosignal lesion in the left hemisphere of cerebellum with extension to CPA and prepontine cistern accompanied by marked peripheral edema and mass effect. Multilobulated enhancement of the lesion is seen in the postcontrast T1-weighted (b) and CT (c) images.

**Figure 4:**
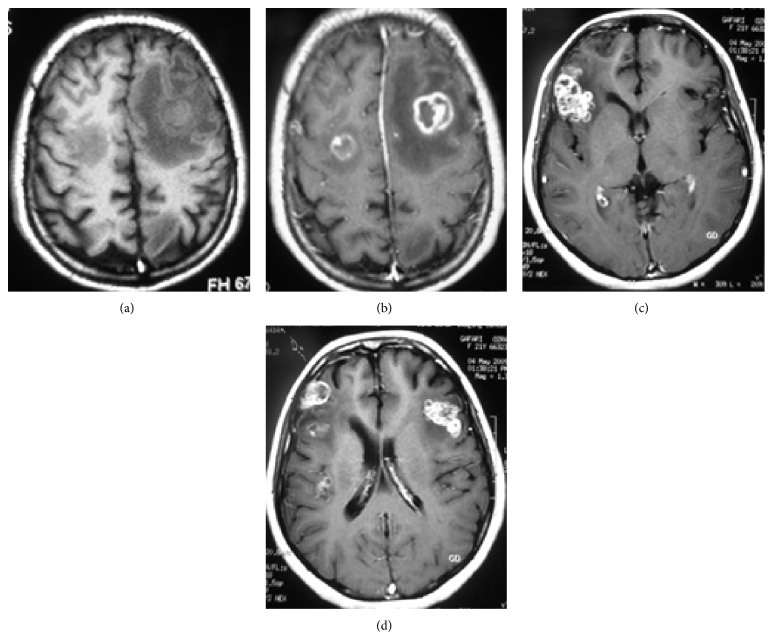
Tuberculous brain abscess. Axial pre- and postcontrast T1-weighted MR images ((a) and (b)) in a 38-year-old male with cognitive and speech disorders show two hypointense lesions in both frontal lobes with peripheral edema, which is severe on left side, and have thick ring-like enhancement (b). (c) and (d) Axial postcontrast T1-weighted MR images in a 22-year-old female with pulmonary miliary tuberculosis and 3-month history of headache, nausea, vomiting, and recent seizure demonstrate bifrontal irregularly enhancing lesions with mild peripheral edema.

Tuberculous abscess is rare and is characterized by a central area of liquefaction with pus. It may be solitary or multiple and is frequently multiloculated (Figure 3) [15]. Tuberculous abscess is different from tuberculomas which contain central caseation and liquefaction mimicking pus. The tuberculous abscess is hypodense with peripheral edema and mass effect on CT. On T2-weighted images, central necrotic area has increased signal intensity. Postcontrast images demonstrate ring enhancement that is usually thin and uniform, although it may be irregular and thick (Figure 4), especially in immunocompromised patients [1, 3, 6, 7, 20, 21].

Magnetization transfer (MT) images improve the conspicuity of all CNS tuberculosis lesions [22–27]. On MR spectroscopy, the peak of amino acids, which could be detected in pyogenic abscess, is not usually seen in tuberculous abscess [22, 27].

### 3.2. Tuberculoma

Tuberculoma is the most common parenchymal lesion in CNS tuberculosis which could be found in any portion of the intracranial space. The lesion may be solitary or multiple and may be seen with or without meningitis. Histologically, the mature tuberculoma is composed of a necrotic caseous center surrounded by a capsule that contains fibroblasts, epithelioid cells, Langhans giant cells, and lymphocytes [28].

On nonenhanced CT scans, tuberculoma may be isodense, hyperdense, or of mixed density. On contrast-enhanced CT, it may present a pattern of ring-like enhancement or, less likely, as an area of nodular or irregular nonhomogeneous enhancement. A central nidus of calcification with surrounding ring-like enhancement, known as the target sign, suggests the diagnosis [19]. Nonenhanced MR studies show a mixed, predominantly low signal intensity lesion with a central zone of high signal intensity and surrounding high signal intensity edema on T2-weighted or FLAIR images [29]. The central high signal intensity zone corresponds to caseating necrosis, and the low signal intensity of the capsule may be related to a layer of collagenous fibrosis with high protein concentration and low water content [29].

**Figure 5:**
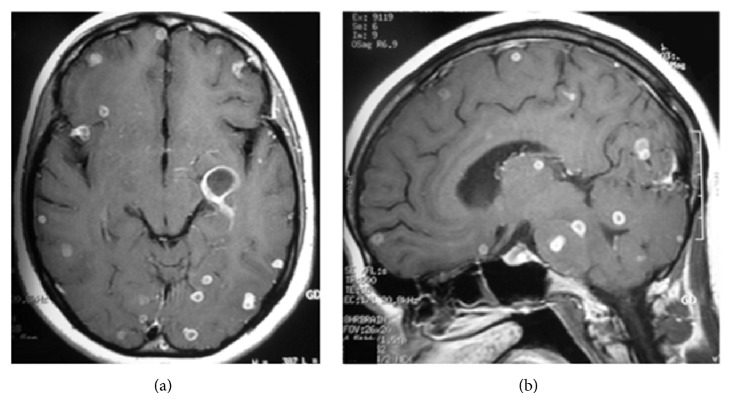
Multiple supra- and infratentorial tuberculomas in a 27-year-old female with history of pulmonary tuberculosis. Tuberculomas are seen as multiple small ring enhancing lesions without peripheral edema in axial and sagittal postcontrast T1-weighted MR images.

Like contrast-enhanced CT, postcontrast MR images usually show a pattern of ring-like enhancement (Figure 5).

Caseating solid granulomas are usually hypointense and strikingly hypointense on T1- and T2-weighted images, respectively. This relative hypointensity is attributed to the granulation tissue and compressed glial tissue in the central core resulting in a greater cellular density than the brain parenchyma. Noncaseating granulomas do not show typical imaging pattern and are usually hypointense to isointense on T1-weighted and hyperintense on T2-weighted images. Homogeneous enhancement is seen after administration of contrast media [7].

Follow-up CT or MR studies are useful in monitoring the response to medical treatment. Paradoxical enlargement of a preexisting tuberculoma or evolution of a new intracranial and spinal tuberculoma in patients receiving adequate treatment may be occasionally seen. However, with continuation of antituberculous therapy, eventual resolution of the tuberculoma usually occurs [29, 30].

**Figure 6:**
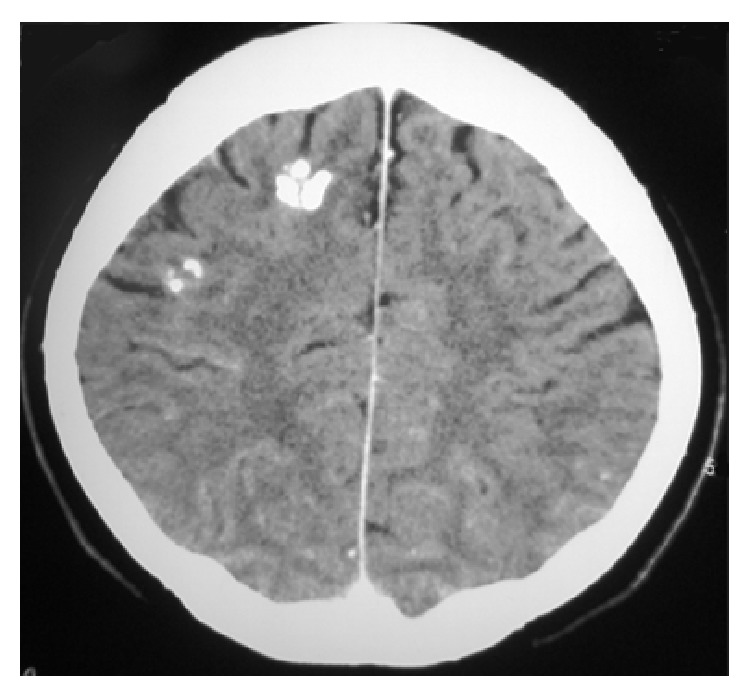
Treated tuberculoma. Axial noncontrast CT image shows two calcified lesions in right frontal lobe without edema or mass effect.

Sometimes, healed tuberculomas appear as calcified foci on nonenhanced CT (Figure 6). Similarly, calcification in the basal cisterns has been demonstrated a few years after meningeal tuberculosis [31].

### 3.3. Miliary Tuberculosis

**Figure 7:**
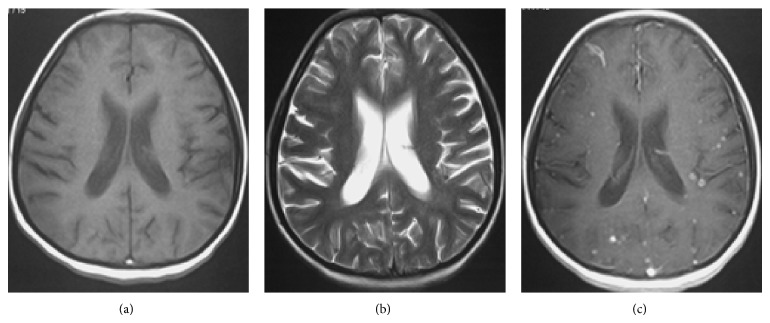
Miliary brain tuberculosis in a 20-year-old female with 3-month history of cough, weight loss, newly added generalized headache, dizziness, nausea, and vomiting. There is no obvious abnormality in the T1- (a) and T2-weighted (b) images. Axial postcontrast T1-weighted MR image (c) shows numerous bilateral tiny enhancing nodules scattered throughout the brain parenchyma.

Miliary tuberculosis is seen mostly in severely immunocompromised patients and is usually associated with meningeal involvement or extracranial primary sites [32]. Since the dissemination is hematogenous, the lesions are usually located at the corticomedullary junctions. The lesions are tiny (2-3 mm in diameter) scattered lesions that may be invisible on noncontrast MR sequences (Figures 7(a) and 7(b)). In visible lesions, MRI shows small lesions that are hypointense on T2-weighted sequences. These lesions occasionally can be seen as small hypodensities on CT scan [13].

Postcontrast T1-weighted MR images show numerous, round, small, homogeneous, enhancing (usually ring enhancement) lesions (Figure 7(c)) [22]. Invisible lesions that may or may not enhance after intravenous injection of gadolinium can be clearly visible on magnetization transfer spin echo T1-weighted imaging with or without contrast [23].

### 3.4. Tuberculous Encephalopathy

Tuberculous encephalopathy typically occurs in young children who may present with convulsion, stupor, and coma with no signs of meningeal irritation or focal neurological deficit. Neuroimaging studies show severe cerebral edema, which may be unilateral or bilateral. Myelin loss in the white matter may result in hypodensity on CT images and hyperintensity on T2-weighted MR images [7, 10, 33].

## 4. Miscellaneous Forms of CNS Tuberculosis

**Figure 8:**
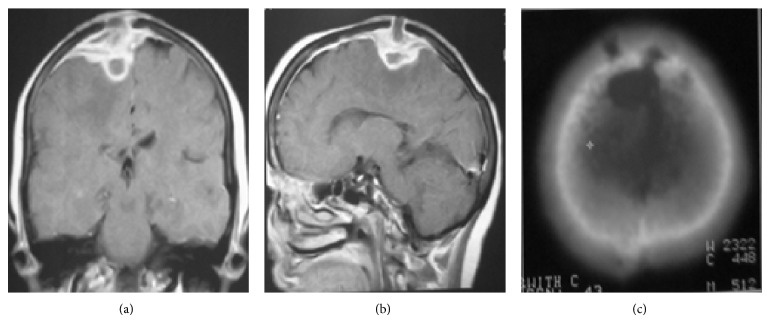
Tuberculous abscess with epidural and subdural empyema and calvarial osteomyelitis. Coronal (a) and sagittal (b) postcontrast T1-weighted MR images demonstrate epidural and subdural collections over the bifrontal cerebral convexities (more on the right side) with intraparenchymal and calvarial extension. Peripheral edema and irregular marked enhancement of the lesion as well as dural enhancement are evident. The bony destructive lytic lesions are seen in the bone window CT image (c).

Osseous and nonosseous spinal/spinal cord tuberculosis, subdural/epidural abscess, and calvarial tuberculosis (Figure 8) are other forms of tuberculosis that may involve CNS with direct or indirect pathways.

Tuberculous spinal meningitis presents on MR imaging as a CSF loculation and obliteration of the spinal subarachnoid space, with loss of the outline of the spinal cord in the cervicothoracic spine and matting of the nerve roots in the lumbar region. Contrast-enhanced imaging reveals nodular, thick, linear intradural enhancement, which may completely fill the subarachnoid spaces [5].

Longstanding arachnoiditis may result in the development of syringomyelia (spinal cord cavitation) that typically demonstrates CSF signal intensity on all MR sequences [5, 29].

**Figure 9:**
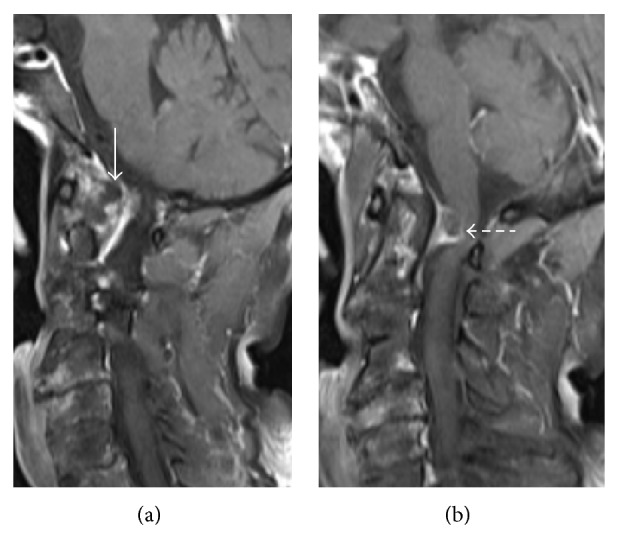
Postcontrast sagittal T1-weighted MR images reveal atlantoaxial tuberculosis with peripherally enhancing epidural abscess (*arrow*) accompanied by cord compression (*dashed arrow*) in a 62-year-old female who presented with gradual bilateral paralysis.

Tuberculous spondylitis results from hematogenous spread of infection to the vertebral body via paravertebral venous plexus of Batson. Typical presentation is involvement of multiple vertebral bodies with sparing of intervertebral disc in early stage and disc involvement in later stages. Paraspinal extension and resultant paravertebral abscess (Pott's abscess) as well as subdural/epidural abscess formation with associated spinal cord compression (Figure 9) are other common findings.

Intracranial subdural or epidural abscess formation may or may not be associated with a primary CNS tuberculous focus and have imaging findings identical to that of other pyogenic abscesses, that is, iso- to hypointensity on T1-weighted images, hyper- or mixed signal intensity on T2-weighted images, and rim enhancement on postcontrast images [29].

## 5. Conclusion

CNS tuberculosis has various imaging appearances, including meningitis, tuberculoma, miliary tuberculosis, abscess, cerebritis, and encephalopathy. In addition, the radiologic manifestations of this disease are not always typical and sometimes may be mistaken with other lesions such as brain tumors. Familiarity with the various imaging presentations of CNS tuberculosis is of key importance for the radiologists and infectious diseases specialists in timely diagnosis, thereby reducing the morbidity and mortality of this potentially life threatening disease.
